# Retracted: Deceptology in cancer and vaccine sciences: Seeds of immune destruction‐mini electric shocks in mitochondria: Neuroplasticity electrobiology of response profiles and increased induced diseases in four generations—A hypothesis

**DOI:** 10.1002/ctm2.389

**Published:** 2021-05-07

The following article from *Clinical and Translational Medicine*, “Deceptology in cancer and vaccine sciences: Seeds of immune destruction‐mini electric shocks in mitochondria: Neuroplasticity electrobiology of response profiles and increased induced diseases in four generations—A hypothesis” by Khatami M, published online on December 19, 2020 in Wiley Online Library (wileyonlinelibrary.com), has been retracted by agreement between the journal Editor‐in‐Chief, Xiangdong Wang, and John Wiley & Sons Australia, Ltd. The retraction has been published because of serious concerns about the quality of the content presented in the article.
